# Flower-visiting insects of genus *Melastoma* (Myrtales: Melastomataceae) at the Fushan Botanical Garden, Taiwan

**DOI:** 10.3897/BDJ.9.e60315

**Published:** 2021-01-26

**Authors:** Joe Chun Chia Huang, Yun Chen Hsieh, Sheng Shan Lu, Wen Chi Yeh, Jia Yuan Liang, Chien Jung Lin, Gene Sheng Tung

**Affiliations:** 1 Botanical Garden Division, Taiwan Forestry Research Institute, Taipei, Taiwan Botanical Garden Division, Taiwan Forestry Research Institute Taipei Taiwan; 2 Forest Protection Division, Taiwan Forestry Research Institute, Taipei, Taiwan Forest Protection Division, Taiwan Forestry Research Institute Taipei Taiwan; 3 Fushan Research Center, Taiwan Forestry Research Institute, Yuan Shan Township, Taiwan Fushan Research Center, Taiwan Forestry Research Institute Yuan Shan Township Taiwan

**Keywords:** buzz pollination, *
Lasioglossum
*, *
Maculonomia
*, *Melastoma kudoi*, sonication

## Abstract

**Background:**

We investigated the diversity and behaviour of insects that visit flowers of four native *Melastoma* (Family Melastomataceae) species of Taiwan and a horticultural hybrid *Melastoma* species at the Fushan Botanical Garden, Taiwan biweekly from May to August 2020. Visits of flower-visiting insects were classified into seven behavioural categories, based on the insects' behaviour and positions on the flower. The data are further assigned into four insect-flower interactions, namely pollination, herbivory, commensalism and neutralism. Our goal is to provide baseline data of insect-plant interactions of *Melastoma*, which is a common, but understudied plant genus in the country.

**New information:**

A total of 1,289 visits to flowers were recorded by at least 63 insect morphospecies belonging to seven orders. The number of insect species recorded per *Melastoma* species ranged from 9 to 39. Visiting, sonication and passing were the three most frequently recorded types of behaviour, collectively accounting for 90.2% (n = 1,240) of the total observations. Pollination was the most dominant insect-flower interaction, accounting for 70.2% of the total observations, followed by neutralism (20.0%), herbivory (6.3%) and commensalism (3.5%). Sweat bees of the genera *Lasioglossum* and *Maculonomia* (Hymenoptera: Halictidae) are considered key pollinators to *Melastoma* species in Fushan Botanical Garden, based on their high number of visits and sonication behaviour. Our study provides the first list of insects that visit the flowers of all Taiwan's known *Melastoma* species and description of their interactions with the plants.

## Introduction

With over 5,000 species, Melastomataceae represents one of the largest Angiosperm families distributed in the subtropical and tropical regions around the world (POWO 2019). Members of this flowering family have a complicated evolutionary history (Renner 1993, Stein and Tobe 1989) and exhibit diverse morphological traits (Dellinger et al. 2018, Renner 1989, Varassin et al. 2008) and reproduction biology (Dellinger et al. 2019, dos Santos et al. 2012, Peng et al. 2014). The diversification of Melastomataceae is partially a result of hybridisation events. Interspecific hybridisation within a genus (Dai et al. 2012, Hawkins et al. 2016) and between genera (Hawkins et al. 2016, Zhou et al. 2020) have been reported. Empirical studies suggest that hybridisation in some genera of Melastomataceae are likely mediated by specialised insect pollinators. The pollination syndrome in Melastomataceae is mainly, but not exclusively, dependent on bees (superfamily Apoidea) that are able to vibrate pollen from poricidal anthers by sonication (Renner 1989). Although interspecific hybridisation via insect pollinators has been observed in Melastomataceae native to Asia, studies on insect-flower interactions in Melastomataceae are largely focused on New World species (e.g. Brito et al. 2016, Brito et al. 2017, Pereira et al. 2011, Renner 1989).

There are 18 species belonging to 12 genera of Melastomataceae in Taiwan (Huang and Huang 1996). Of these, *Melastoma* is the most speciose genus with four species. Two of which, namely *Melastoma kudoi* Sasaki and *M. scaberrima* (Hayata) (previously known as *Otanthera scaberrima*, but see Yang and Liu 2002) are endemic to the Island country, whereas the other two species, *M. candidum* D. Don and *M. malabathricum* L. are widely distributed in Asia, the Pacific and Australia (GBIF Secretariat 2019). Amongst the four species, *M. kudoi* is the rarest species, which has only been recorded from the type locality in central Taiwan. The population of *M. kudoi* is considered highly threatened and included in the national Red List (Editorial Committee of the Red List of Taiwan Plants 2017, listed as *M. intermedia* Dunn, but see the recent taxonomy revision by Dai et al. 2019) due to habitat disturbance and lack of inclusion in protected areas (Huang and Huang 1996). The other three *Melastoma* species can be commonly found in the lowlands up to mid-altitude mountainous areas (Huang and Huang 1996). Despite the great richness of *Melastoma* species in Taiwan, information about pollinators of these species is limited.

To date, only one study on the pollination biology of one *Melastoma* species, *M. candidum*, in Taiwan has been published (Liu et al. 2008). Noteworthy, interspecific hybridisation in this genus is often observed in both wild and cultivated plants in China and Southeast Asia (Cai et al. 2019, Wu et al. 2019, Zhou et al. 2017). Although genetic introgression has not been reported from Taiwan, co-occurrence of congeners, including the endangered and endemic *M. kudoi*, is common in Taiwan (C.J. Lin, unpublished data). Moreover, studies show that the primary pollinators for *Melastoma* species are non-specialised bees (e.g. *Amegilla*, Nomia (Maculonomia) and *Xylocopa* bees for *M. affine* (*M. malabathricum*), Gross 1993; *Bombus*, Nomia (Maculonomia) and *Xylocopa* bees for *M. candidum*, Liu et al. 2008). These generalist bees are also widely distributed in Taiwan (WCY and SSL, unpublished data) and their habitats commonly overlap with *Melastoma* species in the country. Whether these bees would visit all *Melastoma* species remains unknown. Therefore, understanding the pollinator fauna of all *Melastoma* species in Taiwan is essential to protect the *Melastoma* diversity, particularly the two endemic species, from potential genetic introgression. In the present project, we present the first checklist of flower-visiting insects of all known *Melastoma* species in Taiwan, based on empirical data.

## Project description

### Funding

Project of Future Plants

## Sampling methods

### Study extent

**Figure 1.**
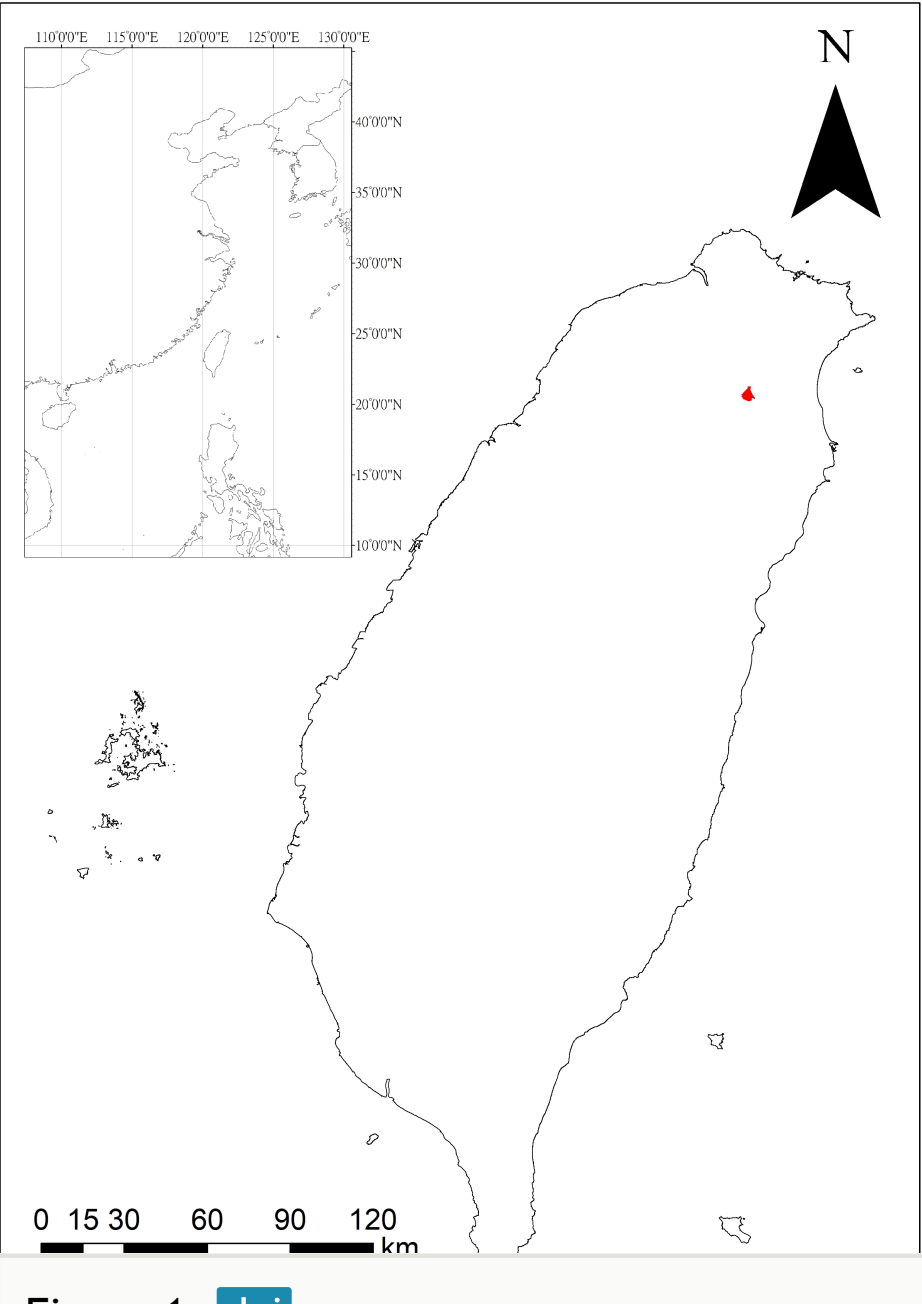
Fushan Botanical Garden, Taiwan. The red polygon delineates the Fushan Experimental Forest.

Established in 1990, Fushan Botanical Garden (FBG) (24°45'21.2"N, 121°35'43.5"E) is located in the mountainous area in the northeast of Taiwan Island (Fig. 1). The garden is part of the Fushan Experimental Forest, which covers approximately 1,098 ha. The vegetation is characterised mainly by natural broad-leaf forest, dominated by trees of the families Lauraceae and Fagaceae (Su et al. 2010). The region has a subtropical monsoon climate and is generally humid throughout the year. The mean temperature of 18.4℃ ranging from 10℃ to 30℃ and peaks in the summer season (June-August). The annual rainfall is 3,787 mm, with more rain during the typhoon season (late August-October) (Lu and Huang 2013). The study was conducted at the garden’s nursery and surrounding trails.

### Sampling description

***Melastoma* flower visiting insect survey**

Data on the diversity of insects that visited the flowers of all *Melastoma* species were obtained biweekly at FBG from 7 May 2020 to 19 August 2020. *Melastoma* species included *M. malabathricum* L., *M. candidum* D. Don, *M. kudoi* Sasaki and *M. scaberrima* (Hayata). We primarily follow the taxonomy of Yang and Liu (2002), but treat *M. septemnervium* as a synonym of *M. candidum* as suggested by the backbone of most catalogues (GBIF Secretariat 2019). *Melastoma malabathricum* is the only species of the four that is native to this region of Taiwan (CJL, unpublished data). For *M. candidum*, we included both the typical purple-flowered form and the white-flowered form. Ten wild *M. malabathricum* individuals were selected along the trails adjacent to the nursery. For the remaining species, including the white flower variant of *M. candidum*, 10-15 planted individuals for each type/species were used from the nursery. Ten planted individuals of a horticultural hybrid of *M. scaberrima* and *M. kudoi* (tentatively named as *Melastoma kudoi* x *Melastoma scaberrima*) were also included. For each survey session, observations of insects were made by 2-4 people at the same time for two consecutive days. The observation began roughly 45 mins after sunrise, usually between 6:30 am and 6:45 am and ended at around 11:30 am when flowers began closing or were out of pollen (JCCH, unpublished data). In the early stage of the study by mid-June 2020, continuous observations were made for *M. malabathricum* in the trails and the rest of the species in the nursery alternatively at 20-min intervals. After the end of *M. malabathricum* flowering season in mid-June, the observations were made continuously for all samples in the nursery site. Additional data, made by random observations outside of the scheduled survey sessions during the weekly phenology suvey (usually one hour in the morning) in another project during the same period, were also included to maximise our understanding of the diversity of flower-visiting insects. Taxa and behaviour (see the next section for details) of insects with body length > 3 mm present on the adaxial surface of flowers were recorded. Insects were identified visually in the field to the finest taxonomy level, whenever possible. For pollinators that could not be identified in the field, 1-3 individuals of each morphospecies were collected using a butterfly net or a plastic bag. All insect species were identified morphologically, following existing keys (Dubitzky et al. 2008, Hsu et al. 2018, Johnson and Triplehorn 2005, Starr 1992).

**Behaviour and insect-flower interaction classification**

**Figure 2.**
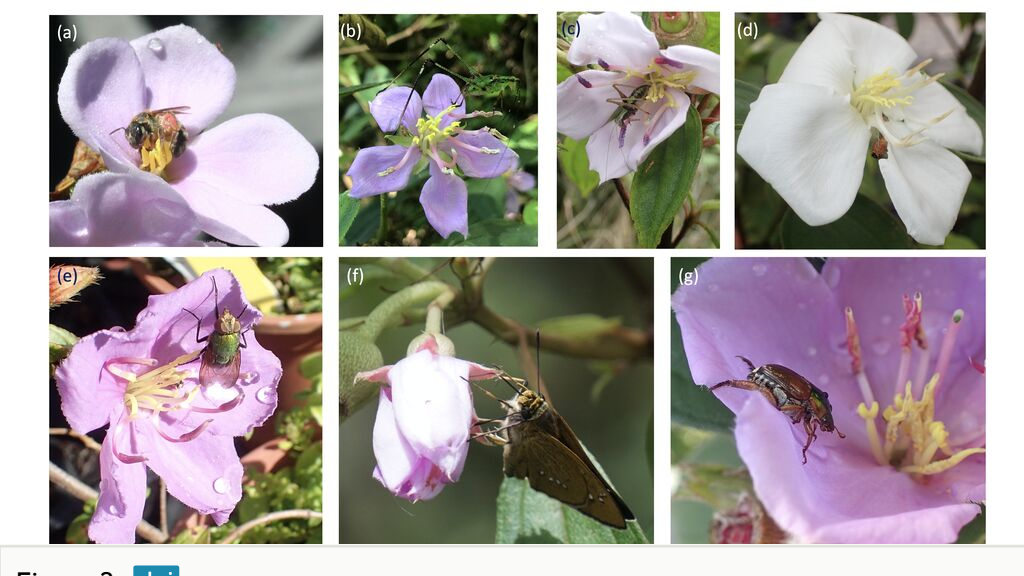
Examples of the seven types of flower-visiting behaviour exhibited by insects on *Melastoma* species: sonication (a), visiting (b), stamen herbivory (c), petal herbivory (d), recycling (e), drinking (f) and passing (g).

Types of behaviour of insects visiting flowers of *Melastoma* species were recorded by direct observations in the field. Further confirmations were made, based on pictures and videos taken using phone cameras. Seven behaviour categories were defined, depending on how insects interact with the flower and the location on the flower where the behaviour occurred, namely sonication, visiting, stamen herbivory, petal herbivory, recycling, drinking and passing (Table 1, Fig. 2). We did not include pollen theft, another important insect behaviour related to interactions with flowers reported in other studies (e.g. Hargreaves et al. 2009). Despite bees often being observed placing their mouth parts at the porous dehiscence of the anther during our observation, there was no evidence that they removed pollen grains from the anthers. In many cases, bees stepped on anthers before they inserted their tongues and then sonicate the anthers afterwards. In other cases, especially near the end of the flowering season or at the last two hours before flowers closed, bees often left the flowers without sonicating the anthers after they performed such behaviour. Therefore, instead of pollen theft, we assume that bees assess pollen capacity of the anthers using both mouth parts and legs before they decide to buzz flowers. Under this context, both types of behaviour were included into the category of visiting. The observations of insect behaviour were further assigned into four types of insect-plant interactions, namely pollination, herbivory, commensalism and neutralism, based upon expected direct effects of each behaviour category for both insects and flowers (Table 1).

## Geographic coverage

### Description

Fushan Botanical Garden, north-eastern Taiwan

### Coordinates

24.755 and 24.755 Latitude; 121.595 and 121.595 Longitude.

## Taxonomic coverage

### Description

63 insect morphospecies belonging to seven orders that are associated with five *Melastoma* plant species, including a horticultural hybrid.

### Taxa included

**Table:** 

Rank	Scientific Name	Common Name
class	Insecta	insect
order	Coleoptera	beetle
order	Hymenoptera	bee, wasp, hornet
order	Diptera	fly, midge
order	Hemiptera	bug, plant hopper
order	Lepidoptera	moth, butterfly, caterpillar
order	Blattodea	cockroach
order	Orthoptera	grasshopper, cricket

## Traits coverage

### Data coverage of traits

PLEASE FILL IN TRAIT INFORMATION HERE

## Temporal coverage

### Notes

2020-05-07 through 2020-08-19

## Collection data

### Collection name

Forest Arthropod Collection of Taiwan

### Specimen preservation method

pinned

## Usage licence

### Usage licence

Creative Commons Public Domain Waiver (CC-Zero)

### IP rights notes

This work is licensed under a Creative Commons Attribution Non Commercial (CC-BY-NC) 4.0 License.

## Data resources

### Data package title

Flower visiting insects of *Melastoma* in Taiwan

### Resource link

https://www.gbif.org/dataset/51b39c28-ce6f-4c7f-ba01-261748411e31

### Alternative identifiers

https://ipt.taibif.tw/resource?r=taiwanmelastomapollinator; 51b39c28-ce6f-4c7f-ba01-261748411e31

### Number of data sets

1

### Data set 1.

#### Data set name

Flower-visiting insects of *Melastoma* in Taiwan

#### Data format

Darwin Core

#### Number of columns

31

#### Description

This resource (Huang et al. 2020) is a summary of the flower-visiting insect occurrence records, based on the observations of this project. The information of flower visiting and flower are addressed in "occurrenceRemarks" and "associatedTaxa", respectively. The dataset is in Darwin Core and published on GBIF.

**Data set 1.** 

Column label	Column description
occurrenceID	An identifier for the Occurrence (as opposed to a particular digital record of the occurrence). In the absence of a persistent global unique identifier, construct one from a combination of identifiers in the record that will most closely make the occurrenceID globally unique.
basisOfRecord	The specific nature of the data record.
eventDate	The date-time or interval during which an Event occurred. For occurrences, this is the date-time when the event was recorded. Not suitable for a time in a geological context.
country	The name of the country or major administrative unit in which the Location occurs
county	The full, unabbreviated name of the next smaller administrative region than stateProvince (county, shire, department etc.) in which the Location occurs.
municipality	The full, unabbreviated name of the next smaller administrative region than county (city, municipality etc.) in which the Location occurs. Do not use this term for a nearby named place that does not contain the actual location.
locality	The specific description of the place. Less specific geographic information can be provided in other geographic terms (higherGeography, continent, country, stateProvince, county, municipality, waterBody, island, islandGroup). This term may contain information modified from the original to correct perceived errors or standardise the description. Comments
minimumElevationInMetres	The lower limit of the range of elevation (altitude, usually above sea level), in metres.
decimalLatitude	The geographic latitude (in decimal degrees, using the spatial reference system given in geodeticDatum) of the geographic centre of a Location. Positive values are north of the Equator, negative values are south of it. Legal values lie between -90 and 90, inclusive.
decimalLongitude	The geographic longitude (in decimal degrees, using the spatial reference system given in geodeticDatum) of the geographic centre of a Location. Positive values are east of the Greenwich Meridian, negative values are west of it. Legal values lie between -180 and 180, inclusive.
geodeticDatum	The ellipsoid, geodetic datum or spatial reference system (SRS) upon which the geographic coordinates given in decimalLatitude and decimalLongitude are based.
coordinateUncertaintyInMetres	The horizontal distance (in metres) from the given decimalLatitude and decimalLongitude describing the smallest circle containing the whole of the Location. Leave the value empty if the uncertainty is unknown, cannot be estimated or is not applicable (because there are no coordinates). Zero is not a valid value for this term.
scientificName	The full scientific name, with authorship and date information if known. When forming part of an Identification, this should be the name in the lowest level taxonomic rank that can be determined. This term should not contain identification qualifications, which should instead be supplied in the IdentificationQualifier term.
kingdom	The full scientific name of the kingdom in which the taxon is classified.
phylum	The full scientific name of the phylum or division in which the taxon is classified.
class	The full scientific name of the class in which the taxon is classified.
order	The full scientific name of the order in which the taxon is classified.
family	The full scientific name of the family in which the taxon is classified.
genus	The full scientific name of the genus in which the taxon is classified.
specificEpithet	The name of the first or species epithet of the scientificName.
infraspecificEpithet	The name of the lowest or terminal infraspecific epithet of the scientificName, excluding any rank designation.
taxonRank	The taxonomic rank of the most specific name in the scientificName.
identificationRemarks	Comments or notes about the Identification.
lifeStage	The age class or life stage of the biological individual(s) at the time the Occurrence was recorded.
vernacularName	A common or vernacular name.
associatedTaxa	A list (concatenated and separated) of identifiers or names of taxa and their associations with the Occurrence.
behaviour	A description of the behaviour shown by the subject at the time the Occurrence was recorded.
fieldNumber	An identifier given to the event in the field. Often serves as a link between field notes and the Event.
catalogNumber	An identifier (preferably unique) for the record within the dataset or collection.
institutionCode	The name (or acronym) in use by the institution having custody of the object(s) or information referred to in the record.
recordedBy	A list (concatenated and separated) of names of people, groups or organisations responsible for recording the original Occurrence. The primary collector or observer, especially one who applies a personal identifier (recordNumber), should be listed first.

## Additional information

**Results**

A total of 1,298 insect visits were observed, which generated 911 occurrence records of flower-visiting insects, of which more than one-third of the visits were made to the horticultural hybrid species, *Melastoma kudoi* x *Melastoma scaberrima* (n = 437). Of the remaining observations, 12-19% were recorded for each of the remaining species/forms and only 3.8% of the observations were recorded for *M. scaberrima*. Around 15.6% and 56.3% of the insects sampled could be identified to species and genus, respectively and the rest are identified to family or higher levels (Table 2). The number of insect taxa recorded from each *Melastoma* species ranged from 9 to 39 morphospecies, for a total across all *Melastoma* species of at least 63 insect morphospecies of seven orders (Table 2).

Visiting, sonication and passing were the three most commonly-recorded types of behaviour, comprising 37.3%, 32.8% and 20.0%, respectively, of the total observations of behaviour (n = 1,240). The other four behaviour categories only accounted for less than 10.0% of the total observations. With 870 observations, pollination was the most dominant insect-flower interaction recorded on *Melastoma* species, followed by neutralism (n = 248), herbivory (n = 78) and commensalism (n = 44).

**Figure 3.**
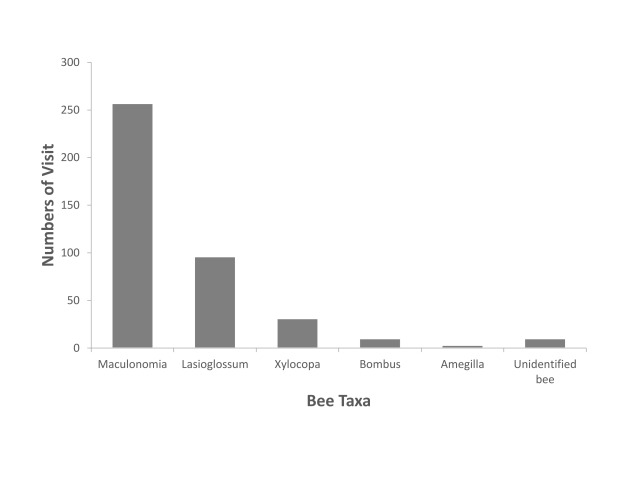
Number of visit by different bee taxa that exhibit sonicating behaviour on flowers of *Melastoma* species.

**Figure 4.**
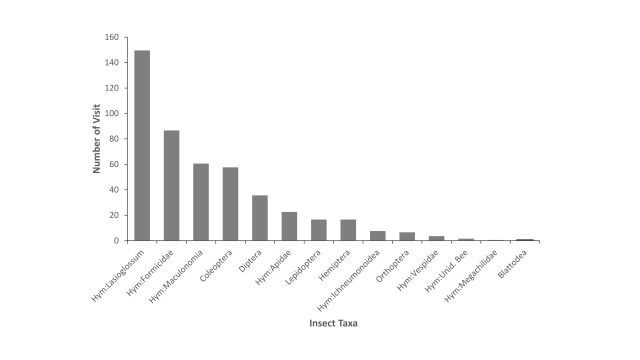
Number of visits by different insect taxa that exhibit pollinating behaviour on flowers of *Melastoma* species. On the x-axis, Hym denotes order Hymenoptera; Unid. Bee denotes unidentified bee.

Pollinating insects that demonstrated sonication behaviour were exclusively bees in families Apidae and Halictidae (Hymenoptera: Superfamily Apoidea). Amongst all sonicating bees, sweat bees of genera *Lasioglossum* and *Maculonomia* were the two most common taxa, accounting for 89% of all flower visits (Fig. 3). There was a higher diversity of pollinator taxa showing visiting behaviour than other types of behaviour on *Melastoma* flowers, including insects of 22 families of all seven orders. *Lasioglossum* and *Maculonomia* bees, adult insects of Coleoptera (mainly families Chrysomelidae and Elateroidea) and Formicidae (Hymenoptera) were the four most frequently encountered taxa in our samples (Table 1Fig. 4).

**Discussion**

This study provides the first checklist of flower-visiting insects to all *Melastoma* species in Taiwan with an emphasis on insect-plant interactions, based on our field observations. Our data show a diverse flower-visiting insect fauna of at least 63 morphospecies which is higher than observations in similar studies on *Melastoma* (Gross 1993, Liu et al. 2008, Peng et al. 2014, Peng et al. 2012). The majority of the insects exhibited sonicating and visiting behaviour, which presumably can be linked to pollination interaction. Buzz-pollinating bees of the families Apidae and Halictidae and particularly members of the genera *Lasioglossum* and *Maculonomia*, were the most common pollinators of *Melastoma* plants in our study site. These findings support the previous conclusion that this genus is primarily buzz-pollinated and highly dependent on bees for pollination.

Despite the commonality in the dependence of buzz-pollinating bees, our results reveal a different bee pollinator composition to other studies on *Melastoma* plants, even for the same plant species. Liu et al. (2008) studied pollination biology of *Melastoma candidum* and other three confamiliar species in Melastomataceae in central Taiwan and found that bees of genera *Bambus* and *Xylocopa* (both Apidae) are the primary pollinators. Studies on *M. malabathricum* (*affine*) in Australia (Gross 1993) reported *Xylocopa*, *Amegilla* (family Apidae) and Nomia (Maculonomia) as the main pollinators. Studies on several *Melastoma* species in southern China suggested that *Bambus* and *Xylocopa*, as well as *Amegilla* bees, are the most important pollinators (Liu et al. 2008, Luo et al. 2008, Peng et al. 2012, Peng et al. 2014). Except *Maculonomia* bees, these common bee pollinators of *Melastoma*, particularly the genus *Amegilla*, represent the minority in our observations.

The discrepancy between studies could be explained by the variations in local bee fauna. Landscape features (Ferreira et al. 2013, Sritongchuay and Bumrungsri 2016), elevation effect (Hoiss et al. 2015) and biogeography (Traveset et al. 2016) could greatly shape bee assemblages and associated pollination networks via trait-filtering resource partitioning and phenological mismatches between pollinators and plants. This might not be the case in this study, because *Amegilla* and *Bambus* bees are both considered common and abundant at the Fushan Botanical Garden (WCY and SSL, unpublished data). For example, *Amegilla* were abundant at the nursery, but rarely visited *Melastoma* flowers throughout the study period (JCCH, YCH, WCY and SSL, unpublished data). A possible cause of the shifted pollination niches is that local bees might not recognise the experimental *Melastoma* plants as an available food resource (Williams 2002) since three of the four plant species are not native to Fushan. Nevertheless, lack of experience cannot completely explain why these bees did not visit the native *M. malabathricum* often. Other studies show that inter-specific competition of pollinators and pollens mediated by floral neighbourhoods (Bruckman and Campbell 2014) and the presence of a super pollinator (Gross and Mackay 1998, Thomson 2004), respectively, could also significantly change the pollinator-plant partnership. Further studies are necessary to clarify the causes of the shifted pollination network in Fushan.

The occurrence of herbivores and their damage to flower structures could supress the pollination process in several ways. First, complete loss of stamens and pollens inside certainly terminate the further chances of pollen transferring. Konzmann et al. (2020) demonstrated that physical modification of anthers could greatly affect the efficiency of pollen-spreading to bees in a neotropical Melastomataceae species. In this case, bees may fail to load pollen to their body if they sonicate damaged stamens, regardless of the amount of pollen remaining inside. Loss and modification of stamen(s) and petal(s) could also reduce the chance of flowers being visited by bees as these floral traits are often found as a resource guide in Melastomataceae plants (Larson and Barrett 1999, Luo et al. 2008). Moreover, in our observations, many herbivorous insects, particularly those with large body size, hindered other flower visitors by active-guarding behaviour or simply covering the reproductive organs with their body (as shown in Fig. 2c). Such trait-mediated processes, mediated by flower herbivores, could also diminish the pollination process at an early stage (Gonçalves-Souza et al. 2008).

While reproduction biology is recognised as an essential part of plant conservation, identifying key pollinators and pollination mechanisms becomes fundamental (Havens et al. 2006, Moza and Bhatnagar 2007). Without such information, cultivation of closely-related species with high hybridisation potential, as observed in *Melastoma* (Dai et al. 2012, Liu et al. 2014, Wu et al. 2019) in *ex situ* collection sites, may increase chances of genetic introgression (Lozada-Gobilard et al. 2020). The hybridisation risk in *ex situ* collections might be more severe for sanctuaries in the tropics as most countries in the tropical regions usually have mega-diverse flora, but often grow high numbers of species in a confined area due to lack of sufficient infrastructure. Noteworthy, Target 8 of the Global Strategy for Plant Conservation aims to preserve at least 75% of threatened species of global flora by 2020. Following the Target, many national and regional botanical gardens, for example, Taiwan Forestry Research Institute, have been expanding their *ex situ* collections since 2012 (Botanic Gardens Conservation International 2012). Further studies on how environmental and ecological factors may drive pollination networks are helpful in preventing *ex situ* plant conservation from accidental hybridisation events.

## Figures and Tables

**Table 1.** The interactions, definitions and expected effects of the seven types of behaviour of insects that were observed to visit the flowers of *Melastoma* species. “+”, “-” and “0” signs denote positive, negative and neutral effects, respectively, of each type of behaviour on the insect (before the left slash) and the plant (after the left slash).

Insect-flower interaction	Type of behaviour	Sign of expected effect	Definition
Pollination			
	Sonication	+/+	Emit buzz sounds when contacting stamens or pistil, producing vibrations that attempt to expel pollen out from anthers
	Visiting	0/0, 0/+	Contact any part of pistil and stamens without consuming and collecting materials and cause no obvious damage to the reproductive organs
Herbivory			
	Stamen herbivory	+/-	Damage stamens, but not the anthers
	Petal herbivory	+/-	Damage petals
Commensalism			
	Recycling	+/0	Consume pollens expelled by other insects from the flower and water, usually on the petals, but occasionally at the female and male organs
	Drinking	+/0	Consume secretion from the flower
Neutralism			
	Passing	0/0	Contact only the petals without consuming and collecting materials and cause no obvious damage to the petals

**Table 2.** Diversity of flower-visiting insects and the accumulative number of visits for each *Melastoma* species.

	***M. malabathricum***	***M. candidum*** **purple-flowered form**	***M. candidum*** **white-flowered form**	***M. kudoi***	***M. scaberrima***	**Hybrid**
** Blattodea **						
** Blaberoidea **						
** Ectobiidae **						
*Symploce* sp.	1					
** Coleoptera **						
** Chrysomeloidea **						
** Chrysomelidae **						
*Arthrotus tricolor*	1					
*Basilepta varians*	3					
*Lagria* sp.	1					
*Monolepta hieroglyphica*						1
*Monolepta signata*			5	5	1	26
*Nonarthra chengi*						1
*Nonarthra* sp.		1				
*Theopea sauteri*	2			1		6
Unidentified leaf beetle	7		4	1		4
** Elateroidea **						
** Elateridae **						
Elateridae gen. sp.	2					
** Scarabaeoidea **						
** Scarabaeidae **						
*Cetoniinae* gen. sp.			3			
*Popillia livida*			4			
*Popillia taiwana*		1	2			
Scarabaeidae gen. sp.	1		2			
** Tenebrionoidea **						
** Mordellidae **						
Mordellidae gen. sp.			1			
Unidentified coleopteran	1		1			
** Diptera **						
** Ephydroidea **						
** Drosophilidae **						
Drosophilidae gen sp.	3					
** Muscoidea **						
** Anthomyiidae **						
*Anthomyia illocata*			1			
** Oestroidea **						
** Calliphoridae **						
Calliphoridae gen. sp.			3	1		13
*Chrysomya* sp.1	5	4	2			2
*Chrysomya* sp.2		3	2	1		3
** Sciaroidea **						
** Sciaridae **						
Sciaridae gen. sp.	2					
** Syrphoidea **						
** Syrphidae **						
*Episyrphus balteatus*	1					
*Paragus* sp.	1					
*Sphaerophoria* sp.	1					
Syrphidae gen. sp.	9		2		1	1
** Tephritoidea **						
** Tephritidae **						
*Spathulina acroleuca*		1				1
Unidentified dipteran	8		4	2	2	8
** Hemiptera **						
** Coreoidea **						
** Coreidae **						
Coreidae sp.	1					
** Fulgoroidea **						
Unidentified planthopper	1					
** Lygaeoidea **						
** Geocoridae **						
*Geocoris varius*	1					
** Miroidea **						
** Miridae **						
*Eurystylus* sp.			1			1
*Pilophorus formosanus*		2	6			
Miridae gen. sp.	2		1			
** Reduvoidea **						
** Reduviidae **	**1**					
Reduviidae gen. sp.	1					
Unidentified bug	5		3			1
** Hymenoptera **						
** Apoidea **						
** Apidae **						
*Amegilla calceifera*	1					
*Amegilla* sp.	5	1	2	1		
*Amegilla urens*	2					1
*Apis cerana*			6			4
*Bombus eximius/flavescens*	3	1	2			4
*Bombus flavescens*	1					
*Ceratina pulchripes*	4		2			
*Ceratina sauteri*					2	
*Ceratina* sp.	4		1	1	1	
*Xylocopa dejeanii sauteri*		1				2
*Xylocopa rufipes*	12					
*Xylocopa tranquebarorum*		8	7	1		4
** Halictidae **						
*Lasioglossum formosae*	8	2	5	1		2
*Lasioglossum scaphonotum*	2					
*Lasioglossum subopacum subopacum*	1	1	1	1	2	
*Lasioglossum* sp.	15	33	92	31	5	105
*Maculonomia planiventris*	1					
*Maculonomia proxima*		2		1		2
*Maculonomia* sp.	1	50	31	63	15	162
** Megachilidae **						
*Megachile rufovittata*	1					
*Megachile* sp.	2					
** Vespidae **						
*Vespa velutina*				2		2
Vespidae gen. sp.			1			
Unidentified bee	11		1		4	
** Formicoidea **						
** Formicidae **						
*Crematogaster* sp.	17	4		1		
Formicidae gen. sp.	22	32	23	27	12	51
*Myrmicinae* sp.		1	1			
*Polyrhachis* sp.	1	1		4	1	7
*Tetraponera thagatensis*	1					
** Ichneumonoidea **						
Unidentified parasitoid wasp		1	7	7		17
** Braconidae **						
Braconidae gen. sp.	1		3	1		1
** Ichneumonidae **						
Ichneumonidae sp.			1			
Unidentified hymenopteran			1			
** Lepidoptera **						
** Arctiidae **						
Arctiidae gen. sp.	1					
** Erebidae **						
*Euproctis* sp.	2	2	7	1		
** Noctuidae **						
Noctuidae gen. sp.	1					
** Papilionoidea **						
** Hesperiidae **						
*Borbo cinnara*			1			
Hesperiidae gen. sp.	1	2	3	1		3
** Lycaenidae **						
Lycaenidae gen. sp.					1	
** Nymphalidae **						
*Athyma selenophora*						1
** Papilionidae **						
*Graphium sarpedon*						1
Unidentified butterfly	9	1	1	3	2	
** Orthoptera **						
Unidentified orthopteran	1		1			
** Acridoidea **						
Unidentified grasshopper	2	5				
** Acrididae **						
*Xenocatantops brachycerus*	3					
** Tettigonioidea **						
Unidentified bush cricket	5					
** Tettigoniidae **						
*Conocephalus melas*	3					
*Mecopoda* sp.	2					
Mecopodinae gen. sp.	3					
